# Welfare of Aged Horses

**DOI:** 10.3390/ani1040366

**Published:** 2011-10-31

**Authors:** Catherine McGowan

**Affiliations:** Institute of Ageing and Chronic Disease, Faculty of Health and Life Sciences, University of Liverpool, Leahurst, CH64 7TE, UK; E-Mail: C.M.Mcgowan@liverpool.ac.uk

**Keywords:** geriatric, human-horse bond, health, management, welfare

## Abstract

**Simple Summary:**

Horses form a unique and special part of their owners' lives and aged horses are no exception. This review considers the health and management of aged horses. In particular how owners manage and care for their aged horses, what diseases and conditions they suffer from and what factors affect their quality of life. As an aged horse reaches the end of its life, an owner will be faced with judging its quality of life and making the decision to end its suffering. The veterinary surgeon plays an essential role in supporting the owner in this process.

**Abstract:**

Horses form a unique and special part of their owners' lives and aged horses are no exception. This review considers the health and management of aged horses, including the role of the owner and their perceptions of aged horses, potential threats or risks to their welfare and finally, factors affecting quality of life and euthanasia of aged horses. Owners of aged horses are concerned about the health, welfare and quality of life of their aged animals. Yet surveys of management and preventive healthcare reflect that there may be some limitations to what owners are actually achieving in practice. They show declining management as horses age, particularly for the retired horse and insufficient appropriate preventive healthcare via veterinary surgeons. The veterinary surgeon plays an essential and influential role in preventive healthcare, management of diseases and disorders and ultimately in the decision making process for euthanasia of aged horses at the end of their lives. The value of aged horses should not be underestimated by veterinarians and others working with them and the continuing care of aged horses should be regarded with the same importance as the care of younger horses with more obvious monetary value.

## 1. Introduction: The Human-Horse Bond and the Aged Horse

The role of horses in western society changed dramatically during the past century with the decline of the working horse and concurrent rise of the performance horse. The various equestrian disciplines, showing and racing industries as well as associated breeding establishments emerged to dominate horse use. However in the past few decades the role of the horse as a companion animal has become more apparent. A survey of over 47,000 households across the USA indicated that 38.4% of horse owners considered their horses to be family members, more than half (56.5%) considered their horses to be a pet/companion, with only 5.1% considering them to be property [1].

Not that this should be surprising. The human-horse bond is unique and powerful and has been for centuries. Cavalry riders once relied on their horses in battle and could owe their lives or the success of a campaign to an exceptional horse. The owner, trainer and rider of a successful racehorse could be indebted to the animal for financial success. Yet the human-horse bond exists across the spectrum of horses and horse owners, not just these elite and exceptional horses. In fact, horse owners in the lowest income bracket and the lowest educational level were more likely to consider their horse as a family member than people of higher income and educational status [1]. There are many factors which are involved, but some of these include the ability to control such a powerful animal, the feeling of superiority or status when riding and the shared experience of exercise, recreation, competitive sport or performance [2,3].

Irrespective of a horse's career, a horse will usually outlive its performance or reproductive capability by more than a decade and sometimes even 2 or 3 decades. Aging has been studied extensively in human [4,5] and canine [6,7] populations with several ways of defining what constitutes “aged”. Overall, aging can be considered to be a generalised impairment of physiological functions, a decreased ability to respond to a wide range of stresses, an increased risk of age-associated disease and an increased likelihood of death [8]. Aging is commonly defined based on physiological aspects of aging. However, aging can also be defined in chronological or demographic terms.

Physiological age refers to the internal function of an animal, once it has reached peak performance and senescence begins [9]. It is generally accepted that a horse has aged beyond its physiological peak at 15 years of age [10]. This age appears to be consistent with deterioration of equine reproductive performance. There are significant increases in collagen fibres in the testes of horses >20 years of age as compared to horses <20 years of age [11] and the number of live foals born in mares declines beyond 13 years of age [12]. However, the functional age of a horse may depend significantly on its use. For example, a Thoroughbred racehorse may be past its physiological peak for racing performance well before 10 years of age, while a grand prix dressage horse may continue to improve its performance after 15 years of age.

Chronological age refers to an animal's numerical age compared to its expected longevity. Horses often live to be in their 30s with some reported to live into their 40s or even longer [13-15]. Therefore, chronological age may overestimate the definition of an aged horse, as the upper quartile of a horse's natural lifespan of 40 years begins at 30 years of age. One of the first reported studies on aged horse health appears to have used chronological age to divide horses in to two groups. They used “old” for the horses aged 20–29 years and “very old” for horses 30 years and older [16]. This age definition was supported by horse owners who reported that they considered a horse to be old at an average age of 22 years [15].

Demographic age is determined by survival relative to that of a population in a region and thus could be limited by economics, use and social issues [9]. The age at which one becomes demographically old is defined as the age at which there is only 25% survivorship at or above that specific age [17]. Mellor *et al.* [18] used 15 years of age as the transition from young horse to an old horse. They found 25% of the population to be over the age of 15 years [18]. It is interesting to note that demographic age corresponds to physiological decline not chronological age and is therefore likely to include economics, use and social issues which influence the natural equine lifespan by euthanasia. Recent research has defined the “aged” or “geriatric” horse as 15 years or over and a horse as very old if it is 30 years of age or older [14,19,20].

The human-horse bond is powerful and enhanced by a long duration of ownership [21] so it is expected that many people will keep an aged horse into retirement to live out its days. Yet despite this expectation, there may be threats to that ideal. Horses are expensive animals to keep, they are susceptible to increased disease and reduced function as they age and may need special care or prolonged medical therapy. It is important to consider the implications of the conflicting pressures faced by owners of aged horses. The aim of this review is to consider the health and management of aged horses, including the role of the owner and their perceptions of aged horses, potential threats or risks to their welfare and finally, factors affecting quality of life and euthanasia of aged horses.

## 2. The Role of the Owner and Their Perceptions of Aged Horses

There is indirect evidence that aged horses are increasing in significance within the equine industry with more aged horses being presented for veterinary care. In a study in the USA, geriatric horse admissions increased from 2.2% of the total equine patient admissions in 1989 to 12.5% in 1999, an almost 6-fold increase in 10 years [15]. This may be attributable to many factors and is likely to result from a combination of increasingly sophisticated veterinary services accompanied by changes in owner willingness to seek out and finance such services. Horse owners may be seeking to maintain the quality of life of their aged horses rather than opting for neglect or euthanasia.

Recent research has supported that owners of aged horses are interested in both the health and welfare of their aged horses [22]. In a survey of 536 owners of horses 15 years and over, more than 90% of owners recorded their perceptions of health issues they considered to be important in horses aged 15 years and over. As well as perceived health issues, many owners reported welfare issues and preventive care as important issues for aged horses. Owners were most concerned about weight loss (maintaining the horse's condition), arthritis/lameness and teeth/dental care. As well as health issues, many owners reported issues pertaining to the welfare or management of aged horses as important with psychological health (horse “feels” cared for), exercise for health (including not excessive exercise) and protection from the environment (rugging, warmth, shelter) most frequently reported [22].

## 3. Management of Aged Horses

The dedication that owners show towards their aged horses has also been demonstrated in surveys of management practices and how these horses were kept. In two studies carried out in South East Queensland (SE QLD) and the North West and Midlands region of the UK (NW UK), owners of aged horses were surveyed with questionnaires completed for over 900 aged horses in each country [19,20]. Despite quite different selection criteria and locations, many practices across both studies were similar.

The median age (interqartile range, IQ and range) was 20.7 years (IQ 17–23, range 15–44 years) [19] and 20 years (IQ 17–24 years, range 15–40 years) [20], with just over 5% over 30 years of age [19,20]. Aged horses tended to be kept in small groups and the median number of horses managed as a group was 2 (IQ range 2–4) [19], IQ 1–4 [20]. The majority of horses in both studies still continued some form of exercise, but in SE QLD 43% of horses 15 years and older were reported to be retired from exercise [19], while 26% from NW UK were retired or companion animals [20]. Despite smaller numbers of retired horses in the UK study, 61% reported a decrease in intensity of exercise with increasing age [20].

The day to day feeding and management reflected owner dedication to aged horses. The majority of aged horses were in good body condition with 30% and 26% of horses overweight (body condition score > 3/5) and 2% and 4.5% underweight (BCS < 2/5) in SE QLD and NW UK, respectively [23,24].

The wearing of a rug implies that owners are closely monitoring and caring for their aged horses. This was the case in both NW UK and SE QLD, despite quite different climactic conditions. Many horses in SE QLD may have been rugged for protection against biting insects instead of for warmth as may have been the reason in NW UK. In SE QLD, 51% of horses wore a rug ‘usually’ or ‘all of the time’ and only 20% had not worn a rug in the previous 12 months. In contrast, retired horses were less likely to wear a rug [19]. In NW UK, the majority of horses were rugged, especially during the autumn and winter months.

Similarly supplementary feeding implies that owners are closely monitoring and caring for their aged horses. In SE QLD, 71% of aged horses received supplemental feeding or additives to the diet, however, both supplemental feeding and additives decreased with retirement [19] indicating that as a horse ages and becomes retired these may not continue to the same degree, even though they could be potentially more warranted. In NW UK, all aged horses received supplementary forage which probably represents management practices in the UK [20]. However, over half received haylage which is greater than reported in younger horses [18,25] and 40% of owners reported making major changes to their horse's diet as they had aged with an increase in the use of commercial veteran/senior diets and complete mash feeds in older horses [20].

As well as attention to rugging and supplementary feeding, owners in both SE QLD and NW UK paid attention to deworming and hoof care. Almost all owners in both studies had attended to their horses' hooves or dewormed them in the previous year. However, the frequency of hoof care visits per year decreased with retirement [19,26] and the frequency of anthelmintec administration per year reduced with both age and retirement [19]. While retirement could conceivably correspond with a reduced requirement for hoof care, the reduced immune function of aged horses would not support reduction in anthelmintec use [27,28].

Despite their attention to the management of their aged horses in terms of diet, rugging, hoof care and parasite control, owners of aged horses were less diligent when it came to veterinary visits, especially those in Australia where only 40% horses had seen a veterinarian in the previous 12 months and the figures were worse in rural areas compared to urban areas [19]. Similarly dental visits had only occurred in 67% of horses the previous year and only about half had been vaccinated in the previous year and the likelihood of either a dental visit or vaccination in the previous 12 months was reduced for retired horses [19]. While horses is the NW UK were more likely to have had a veterinary visit (82%), dental visit (74%) or vaccination (83% tetanus, 63% influenza) in the previous year, the age of unvaccinated horses was significantly higher than vaccinated horses and vaccination was reduced in retired horses [26]. Again, the reduced immune function of aged horses would not support reduction in vaccination in both studies with age and retirement [29].

## 4. Aged Horse Health

Until recently, our knowledge of aged horse disease was limited to what was reported as being seen in large referral hospitals or from surveys of post-mortem data. Horses represented in referral hospital populations would have had a disease serious enough and economically justifiable to warrant presentation to a veterinary surgeon followed by referral to the university hospital, yet not serious enough to warrant euthanasia by the primary veterinarian. Again, post-mortem data will reflect a subset of horses valuable enough to warrant the cost of post-mortem evaluation and will usually reflect horses previously hospitalised or under treatment by larger practices rather than the population as a whole. However, recently, two large studies have investigated diseases and conditions of aged horses in the field and some important differences have emerged.

In aged horses presented to a university referral hospital, disease occurrence was investigated using retrospective analysis of the primary problem by body system [16]. The study also focussed on the presenting complaint rather than coincidental findings. The most common reasons for admission of 467 horses aged 20 years or greater (by body system involved) were gastrointestinal tract (54%), musculoskeletal (24%) and respiratory (16%) and colic was the overall leading reason for admission. The endocrine, ocular, cardiovascular, skin, reproductive, neurological, urinary and haemolymphatic systems were less commonly involved. Among the 467 horses in the study, the six most common specific diagnoses included pituitary pars intermedia dysfunction or PPID (46/467; 10%), strangulating lipoma of the small intestine (32/467, 7%), laminitis (30/467, 6%), heaves (30/467, 6%), large colon impaction (26/467, 5.5%) and gastric ulceration (26/467, 5.5%) [16].

A brief report on post-mortem examinations performed on 817 horses aged 15 and older over 5 years showed that the most common body system implicated as resulting in death was gastrointestinal, followed by the musculoskeletal and reproductive systems [30]. The high number of reproductive disorders with an increased representation of uterine artery rupture is likely to be due to the survey being undertaken in the breeding region of Kentucky. The author divided the ages into 4 groups: 15–19 years of age, 20–24 years of age, 25–29 years of age and over 30 years of age. When analysing the last two age groups, the most common individual diagnosis was neoplasia and pituitary adenoma (PPID) was the most common of these [30].

In two studies carried out in SE QLD [23] and NW UK [24] examining a selection of horses in the field, the results reveal a very different picture from the studies above. Instead of a predominance of gastrointestinal, musculoskeletal and reproductive emergencies, more chronic conditions were more commonly observed in veterinary examinations in the field. Despite quite different selection criteria and locations, the prevalence of disorders in both studies was remarkably similar.

In SE QLD [23], owners of 974 horses ≥15 years of age were recruited via an equestrian association. From these, 347 horses (35%) were selected for veterinary clinical examination. Dental abnormalities were identified in 96% of horses; with a lower frequency in the incisor region (36%) than the upper cheek teeth region (94%). Dermatological abnormalities were present in 40% which included 14% hirsutism, 11% skin tumours and 11% culicoides hypersensitivity. Ophthalmic lesions frequently indentified were cataracts in one or both eyes (33%) and senile retinopathy (29%). Cardiac murmurs occurred in 43%. Some form of nasal discharge was present in 7% and 21% had abnormalities on pulmonary auscultation at rest. Lameness in at least one limb was detected in 50%; 33% were lame in the forelimbs, 25% were lame in the hind limbs, 2% were lame in all four limbs. Hoof abnormalities were detected in 69%. Age was a significant risk factor for the occurrence of many of the conditions found [23].

In NW UK [24], owners of 908 horses ≥15 years of age were recruited via veterinary practices. From these, 200 horses were selected and for veterinary clinical examination. Dental abnormalities were identified in a very similar proportion of horses (95.4%) as in SE QLD and the cheek teeth were most commonly affected, with cheek teeth diastemata, excessive wear/cupped out teeth and focal overgrowths the most frequently identified conditions. Seventy-one percent had a dermatological abnormality and 22% displayed hirsutism or abnormal moulting. Ophthalmic lesions frequently indentified were vitreous degeneration (66.0%), cataracts (58.5%) and senile retinopathy (33.7%). The prevalence of cardiac murmurs was 20%. Seven percent of horses had a spontaneous cough during the examination, although 18.5% had some form of nasal discharge and 22% had abnormalities on thoracic auscultation at rest. When assessed at walk, 18.6% were lame on at least one limb, while 50.5% were lame in trot. The majority of animals (83.5%) had a reduction in range of motion in at least one joint. Eighty percent of horses had hoof abnormalities [24].

A consistent finding from both studies was that owners reported a much lower frequency of clinical signs or known diseases or disorders than were found on veterinary clinical examination [22-24,31]. Only 35% and 31% of owners from SE QLD [22] and NW UK [26], respectively, reported a known disease or disorder affecting their horses, indicating that many clinical signs were overlooked. In many cases, owners recognised clinical signs of disease when asked about them specifically, but the diseases or disorders associated with the signs their horses were demonstrating had not been diagnosed or treated. For example, hirsutism, which is likely associated with undiagnosed PPID, was identified in 14% and 22% of horses by owners in SE QLD or NW UK. Yet PPID was reported as a known disease or disorder in only 1.6% and 3.3% of these horses respectively. Owners were either not seeking a diagnosis when horses were exhibiting clinical signs or they were seeking a diagnosis from a source that could not provide one.

Similarly, preventive healthcare, especially veterinary visits, dental care, vaccination and hoof care did not correspond to the prevalence of disorders that could be managed by appropriate care [19,26]. This is in contrast with other indicators of owner intent mentioned above regarding management of their aged horses and may imply a lack of appropriate education or information from appropriate sources. It is interesting to consider that the dedication horse owners demonstrate towards their aged horses is largely reflected in management, feed additives and dietary alterations which are not necessarily required. In fact, many aged horses were overweight, very few underweight [23,24] and it has been reported that aged horses in good body condition do not require dietary alteration and that many of the supplements given to aged horses are as yet unproven in their effectiveness [32]. If some of this attention could be diverted to receiving the appropriate education and professional care, especially from veterinary surgeons, then preventive healthcare and management of chronic diseases and disorders of aged horses may be more effective.

The findings from these field studies clearly indicate a number of more chronic conditions which can adversely affect the welfare of aged horses, especially chronic lameness, dental disorders, hoof abnormalities, respiratory and dermatological disease. This combined with the fact that owners may not be able to recognize some of the signs of diseases and disorders indicate the importance of veterinary involvement in management and preventive health of aged horses and the education of their owners.

## 5. Factors Affecting Aged Horse Quality of Life and Euthanasia

Apart from the threats posed to aged horses from chronic disease, potentially insufficient preventive health care and perhaps misdirected dedication by horse owners, at some point aged horses will have a declining quality of life and owners of aged horses will be faced with the issue of euthanasia. There are conflicting and complex relationships between the personality of the owner, the strength of the human-horse bond and the experiences of euthanasia in the past which may affect this decision. Certainly owners can be as guilty of compromising welfare by delaying euthanasia due to fears about doing the right thing or the perceived distress it will cause them or their family, as those owners who compromise welfare by simply neglecting their aged horse.

Many factors influence an owner's decision to euthanase a horse. In a survey of owners of aged horses, owners were asked to rank the importance of factors that may affect their decision. The highest ranked factors included including a hopeless prognosis and conditions causing incurable pain, chronic or recurrent pain or acute severe pain as well as veterinary advice. However, just as highly ranked were the relationship with the horse and anticipated quality of the horse's life [33] further emphasising the concern owners of aged horses have towards the welfare of their horses and the human-horse bond. In another study, similar results were found with veterinary advice, hopeless prognosis, poor quality of life and long-term or painful conditions being most frequently reported [34]. However, owners were asked to identify the most important factor rather than rank a list of factors as in the study above [34]. Both studies highlight the essential and influential role of the veterinary surgeon in the decision making process of euthanasia and explains in part, why the owner looks to the veterinary surgeon for support during and following euthanasia [35].

Quality of life of an aged horse is influenced by many factors, not just health and a recent study has shown that owners of aged horses are well placed to assess the quality of life of their own horses [34] and they perceive many factors as affecting the quality of life of their horses, including nutrition, comfort, company of other horses and exercise regime [34]. Owners of older horses considered nutrition, grooming, comfort and being pain-free to be more important factors, while owners of younger horses reported exercise as a more important factor influencing their animal's quality of life [34]. Owners of horses with a known disease or disorder ranked freedom from pain as higher than owners of horses without a known disease or disorder. In all cases chronic disease causing chronic pain was considered an important negative factor for quality of life in horses [34]. In this study a series of Likert-type questions were developed where owners could rank their horse's quality of life daily in aspects such as overall satisfaction with the horse's general health and quality of life, the extent to which activities are limited by pain or age, interactions with horses or other people, activity and appetite. What was interesting was that in a subsequent paper by the same authors, these Likert questions, as well as clinical signs of disease, horse signalment, demographic and management factors were all analysed as risk factors for mortality using a multivariable model and three of the quality of life Likert factors were retained as significant risks for mortality out of a nine factor multivariable model [36]. Those factors were “extent to which pain limits normal activities”, “has frequency of lying down changed with age” and “is horse able to lie down or roll and get up again easily” [36]. Veterinarians, if engaged in assessing quality of life, should not overlook the role of the owner and the questions identified by these studies.

Euthanasia of horses is a particularly stressful procedure, especially in an emergency situation. Horses are large, powerful and unpredictable, especially when in acute pain or distress. Even during a relatively calm and uneventful euthanasia of a horse it can be shocking for an owner to observe such a powerful animal fall to the ground [37]. However, a recent study found that degree of distress to owners caused by the loss of an aged horse was greater on a visual analogue scale than the degree of distress caused by the actual procedure of euthanasia [33]. This supports research that has shown that owners commonly feel prolonged grief after euthanasia of their horse, comparable to that experienced by the loss of companion animals [38]. Furthermore, the grief index was higher in those respondents who spent a longer period of time per day with their horse [38].

In addition to the human-horse bond, duration of ownership and position a horse might have within a person's daily life or routine [39], the personality of the horse owner can affect the degree of distress or grief felt following euthanasia of their horse. The personality of horse owners who found the decision to euthanse an aged horse particularly hard scored higher in the neuroticism domain of a five-factor personality model than those who found the decision a major but less difficult decision [33]. This might indicate those owners who found the decision more difficult had a greater sense of apprehension, a tendency to experience states of frustration and bitterness and would be susceptible to experiencing guilt, sadness and loneliness [33]. Identifying owners susceptible to such negative feelings is vital and an important responsibility of veterinary surgeons performing euthanasia. There is now evidence that improvement in communication by the veterinary surgeon before, during and after the actual event of euthanasia can help support owners, especially those who are finding the decision very difficult [35,38].

## 6. Conclusions

Horses form a unique and special part of their owners' lives and aged horses are no exception. Owners of aged horses are concerned about the health, welfare and quality of life of their aged animals. Yet surveys of management and preventive healthcare reflect that there may be some limitations to what owners are actually achieving in practice, with declining management as their horse ages, particularly retired horses and insufficient appropriate preventive healthcare via veterinary surgeons.

A very high proportion of aged horses in the field suffered from chronic disease, especially chronic lameness, dental disorders, hoof abnormalities, respiratory and dermatological disease, yet the majority of affected horses were not undergoing veterinary treatment. Many conditions were preventable or manageable and owners of aged horses should be encouraged to seek the advice of veterinary surgeons for annual health checks and preventive care plans for their aged horses. Further, veterinary surgeons should be informed about conditions specific to aged horses. Future research on the development and trialing of an aged horse annual health plan and its impact on undiagnosed and unmanaged chronic disease in aged horses may be warranted.

The human-horse bond was also an important factor in the degree of grief experienced by owners of aged horses and loss of an aged horse was perceived as very distressing by their owners. Owners who found the decision to euthanase their aged horse very difficult were more likely to suffer even more grief due to their personality. The veterinary surgeon plays an essential and influential role in the decision to euthanase a horse and this also places the veterinary surgeon in a position of responsibility to ensure the appropriate communication and support for a grieving owner.

The value of aged horses should not be underestimated by veterinarians and others working with them and the continuing care of aged horses should be regarded with the same importance as the care of younger horses with more obvious monetary value.
